# Predictive Value of the Atherogenic Index of Plasma (AIP) for the Risk of Incident Ischemic Heart Disease among Non-Diabetic Koreans

**DOI:** 10.3390/nu13093231

**Published:** 2021-09-16

**Authors:** Julie J. Kim, Jihyun Yoon, Yong-Jae Lee, Byoungjin Park, Dong-Hyuk Jung

**Affiliations:** 1 Department of Family Medicine, Yonsei University College of Medicine, Seoul 03722, Korea; juliejkim@yuhs.ac (J.J.K.); ghyunyoon@yuhs.ac (J.Y.); 2 Department of Family Medicine, Gangnam Severance Hospital, Yonsei University College of Medicine, Seoul 06273, Korea; ukyjhome@yuhs.ac; 3 Department of Family Medicine, Yongin Severance Hospital, Yongin 16995, Korea

**Keywords:** atherogenic index of plasma, triglycerides, cardiometabolic risk, cohort study, ischemic heart disease

## Abstract

The atherogenic index of plasma (AIP), composed of triglycerides and high-density lipoprotein cholesterol, is a novel critical marker for assessing the risk of atherogenicity and cardiometabolic health. We aimed to prospectively study the association between AIP and incident ischemic heart disease (IHD) risk in a large cohort of non-diabetic Korean adults. Data were assessed from 17,944 participants without diabetes from the Health Risk Assessment Study (HERAS) and Korea Health Insurance Review and Assessment (HIRA) data. The participants were divided into four groups according to AIP quartiles, calculated as log (triglyceride/high-density lipoprotein cholesterol). We prospectively assessed hazard ratios (HRs) with 95% confidence intervals (CIs) for IHD using multivariate Cox proportional-hazard regression models over a 50-month period that followed the baseline survey. During the follow-up period, 332 participants (1.9%) developed IHD. HRs of IHD for AIP quartiles 2–4 were 1.58 (95% CI, 1.03–2.43), 1.82 (95% CI, 1.20–2.78), and 2.11 (95% CI, 1.37–3.24) after adjusting for age, sex, body mass index, smoking status, alcohol intake, physical activity, mean arterial blood pressure, fasting plasma glucose, high-sensitivity C-reactive protein level, and hypertension medication. Higher AIP levels may precede and predict the development of IHD in non-diabetic Korean adults.

## 1. Introduction

In the early twenty-first century, cardiovascular disease (CVD) has become the lead cause of death. Among CVDs, ischemic heart disease (IHD) affects as many as 126 million people, accounting for an estimate of 1.72% of the global population [1,2,3]. IHD is also the leading cause of death in Korea and has increased gradually over the last decade with rapid population aging [4]. Thus, the emphasis should be placed on screening high-risk individuals and taking appropriate preventative measures to control the occurrence of IHD events.

Among conventional risk factors of CVD, an abnormal plasma lipid profile is considered to be an important risk factor for IHD [5,6,7,8]. Clinical emphasis was placed on elevated triglyceride (TG) levels because high TG was found to be an independent risk factor for IHD in previous epidemiological studies [9,10,11,12]. Increasing TG affects the low-density lipoprotein cholesterol (LDL-C) particle phenotype to be smaller, denser, and more easily oxidized, elevating atherogenic potential [13,14,15]. Additionally, the TG and IHD relationship is known to be mediated partly by the concentration of high-density lipoprotein cholesterol (HDL-C) because, within the high TG concentration range, decreasing HDL was shown to be associated with an increased risk of IHD [14,16,17,18,19]. The central lipid parameters: TG and HDL-C are utilized in a calculated value called the atherogenic index of plasma (AIP). AIP has been gaining prominence as a screening tool for dyslipidemia and is regarded as a major cardiometabolic risk factor [20]. The elevated AIP has been shown to play an important role in the pathophysiology of both prehypertension and prediabetes [21,22,23]. Additionally, studies have shown that non-diabetic individuals with IHD had worse prognosis than diabetic patients without IHD [24,25].

This prompts the need for early detection of cardiometabolic risks that can contribute to the development and progression of IHD. To our knowledge, information on the association between AIP and IHD is limited. In this prospective study, the association between AIP and IHD incidence was investigated within a large-scale, non-diabetic adult cohort exported from the National Health Insurance Service data. 

## 2. Materials and Methods

### 2.1. Study Design

**Figure 1:**
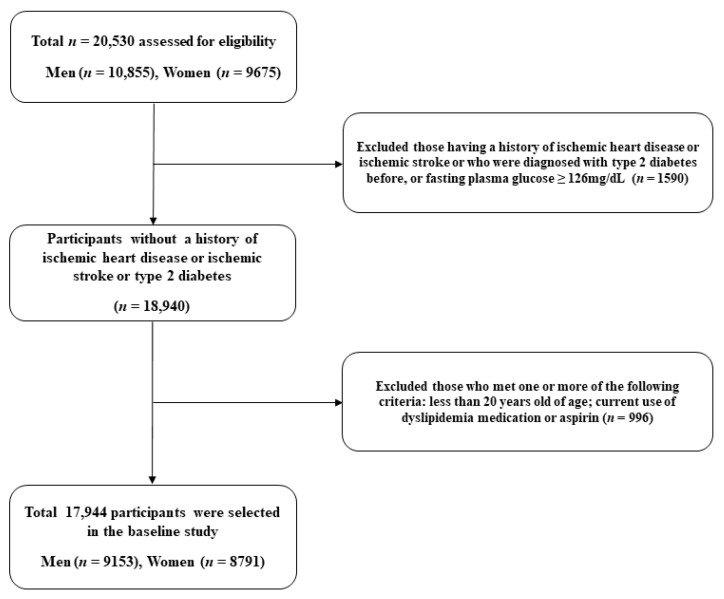
Study population selection.

The present study is a derivative of the Health Risk Assessment Study and Korea Health Insurance Review and Assessment Service (HERAS-HIRA) cohort conducted between November 2006 and June 2010 [26]. The dataset was composed of 20,530 volunteers sequentially visiting for health examination screenings at the Gangnam Severance Hospital of the Yonsei University Health System. Participants meeting any of the following criteria were excluded: a history of IHD; a history of ischemic stroke; previously diagnosed or newly diagnosed diabetes [27]; dyslipidemia medication; or current use of aspirin (Figure 1).

We examined health behaviors and past medical history through structured questionnaires for every participant. The medical staff measured systolic blood pressure (SBP) and diastolic blood pressure (DBP) with arms supported at the heart level, sitting position using mercury sphygmomanometers after 10 min of rest (Baumanometer, Baum Co Inc., Copiague, NY, USA). We defined hypertension as SBP ≥ 140 mmHg, DBP ≥ 90 mmHg, a history of hypertension previously diagnosed by a doctor, or current use of antihypertensive medication [28]; defined impaired fasting glucose (IFG) as a fasting plasma glucose (FPG) from 100 to 125 mg/dL [29]; defined metabolic syndrome as at least three of the following medical conditions: body mass index (BMI) ≥ 25.0 kg/m^2^, SBP ≥ 130 mmHg, DBP ≥85 mmHg, or use of blood pressure-lowering drugs; FPG levels ≥ 100 mg/dL or use of antidiabetic agents; TG ≥ 150 mg/dL; and HDL-C < 40 mg/dL for men and <50 mg/dL for women [30]. The formula for calculating AIP was as follows: the base-ten logarithm of the plasma concentration ratio of TG to HDL-C [31].

### 2.2. Outcomes

The outcomes were acute myocardial infarction (ICD-10 code I21) or angina pectoris (ICD-10 code I20) since the study enrollment [26]. We conduct outcomes assessment over the 50 months since the initial registration by linking each unique 13-digit identification number to the HIRA database.

### 2.3. Statistics

We divided the AIP values into quartiles and compared the clinical characteristics at the baseline. To evaluate the cumulative IHD incidence, we used Kaplan–Meier curves with the log-rank test. We performed multivariate Cox proportional-hazard regression models to assess hazard ratios (HRs) with 95% confidence intervals (CIs) for IHD after adjusting for potential confounding factors, using SAS version 9.4 (SAS Institute Inc., Cary, NC, USA).

## 3. Results

Table 1 shows the baseline characteristics of the study population (*n* = 17,944; 9153 men and 8791 women) based on the AIP quartiles. The mean age, HDL-C, TG, and AIP values were 44.7 ± 10.5 years, 1.4 ± 0.9 mmol/L, 1.4 ± 0.3 mmol/L, and −0.06 ± 0.29, respectively. The subjects with the highest AIP quartile showed the highest BMI, mean arterial pressure, fasting blood glucose, hsCRP, and total cholesterol levels, as well as the lowest mean HDL-C levels. Additionally, this group had the highest proportions of alcohol drinkers and current smokers, as well as the lowest proportion of individuals involved in regular exercise. The incidence of impaired fasting glucose, hypertension, and metabolic syndrome were 17.2%, 19.7%, and 11.5%, respectively, which gradually increased according to the AIP quartiles.

**Figure 2:**
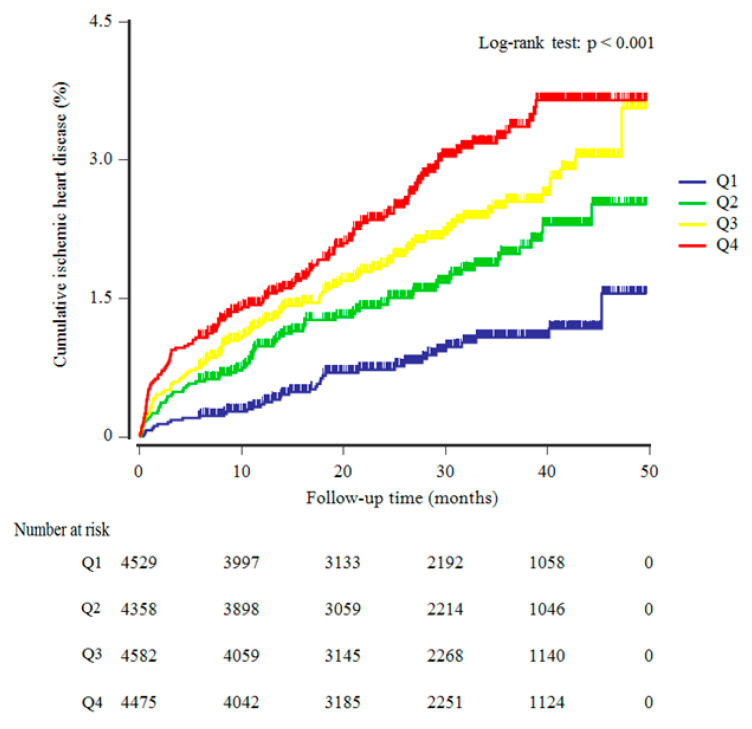
Kaplan–Meier plots indicating the cumulative ischemic heart disease (IHD).

Table 2 shows the overall incidence of IHD according to AIP quartiles during the 50-month follow-up period. During this time, 332 (1.9%, 332/17,944) subjects developed IHD. The IHD incidence rate (per 1000 person-years) was positively related with AIP quartiles. The higher AIP quartiles showed a significantly increased cumulative IHD incidence over the follow-up period after the baseline survey (log-rank test, *p* < 0.001) (Figure 2).

Using univariate and multivariate Cox proportional hazards regression analysis, AIP was correlated with new onset IHD in Table 3 and Table 4. Compared to the HRs of the first AIP quartile (which was used as a reference), the HRs of new onset IHD for the second, third, and fourth quartiles increased proportionately with the degree of AIP. After adjusting for age and sex in Model 1, the HRs for incident IHD were 1.63 (95% CI, 1.10–2.42), 1.85 (95% CI, 1.27–2.71), and 2.34 (95% CI, 1.61–3.40) in the second, third, and fourth AIP quartiles, respectively. Likewise, these longitudinal positive relationships were observed in both Models 2 and 3, where additional adjustments were made for BMI, current smoking, drinking, regular exercise, mean blood pressure, FPG, high-sensitivity C-reactive protein levels, and hypertension medication. The adjusted HR for the highest versus lowest AIP quartile was 2.11 (95% CI, 1.37–3.24) in Model 3. These associations were similar among the male individuals but less prominent than the entire population (Table 5).

## 4. Discussion

This study revealed that elevated AIP values had positive and independent association with the IHD incidence. After making adjustments for potentially confounding factors, the results clearly demonstrated the linear association between an elevated AIP and the risk of IHD.

Several surrogate markers of dyslipidemia for predicting atherosclerosis and CVD have been investigated in clinical practice. Thus far, high fasting TG is the strongest independent risk factor for IHD at all levels of HDL-C. Within the high TG level, high HDL-C levels showed lower IHD risk compared to those with low HDL-C levels. This meant that the high-TG and low-HDL-C concentration was the strongest risk factor for IHD, while low-TG and high-HDL-C concentrations had a three times lower risk of IHD [13]. Similarly, our study also showed a trend of increasing TG and decreasing HDL-C as the incidence of IHD increased. This finding is biologically plausible because increasing TG levels cause an elevated production of TG-rich atherogenic lipoproteins, thus linearly increasing the IHD risk despite consideration of the anti-atherogenic effects of HDL-C. AIP, a calculated value that utilizes the two central lipid parameters, reflects the distribution of particle sizes in lipoprotein subclasses, making AIP a great surrogate and independent predictor of atherosclerosis and all-cause mortality [32,33,34,35]. Wu et al. also found that AIP is a superior predictor of the coronary artery disease (CAD) risk than isolated levels of lipid parameters [36]. In addition, AIP also correlates significantly with the traditional risk factors for atherosclerosis and the CAD severity [36,37]. This mechanism may explain why isolated hypercholesterolemia had no correlation to a high risk of IHD; instead, the relevance of AIP was shown to be higher concerning the risk of IHD.

Accumulating evidence supports the role of AIP as a predictive index for both CVD and metabolic syndrome (MetS) and type 2 diabetes [38,39]. MetS is a syndrome of multiple abnormalities in glucose and lipid metabolism which induce hypertension, dyslipidemia, glucose intolerance, and insulin resistance (IR) [40,41,42,43]. Many risk factors for MetS are similar to those of IHD [44]. This was confirmed by many epidemiological studies that revealed the association and showed that MetS presence predicted the risk of IHD in the clinical field [45,46]. This trend of association was also observed in our study, where metabolic syndrome increased with increasing AIP quartiles. Due to the well-established association between IHD and diabetes, it is also important to examine how AIP is related to well-known markers of IR in the presence of IHD. A well-established index of both CVD and IR is the triglyceride-glucose (TyG) index, the natural logarithm (Ln) of the product of plasma glucose and TG. The TyG index has been suggested to have a positive association with a higher prevalence of CVD, and it has also been used in healthy adults as a surrogate of IR [47,48]. Similar to the TyG index, AIP has recently been considered to be an independent predictor of subclinical coronary artery disease [49]. Since TG and HDL-C levels can be used as the basis for identification of IR and IHD, elevated AIP can predict the trends of common risk factors such as low physical activity, high BMI, hypertension, type 2 diabetes, and elevated FPG levels. Several studies indicate that atherosclerosis-related adverse events frequently occur in populations with low CV risk burden as well as those with high CV risk [50,51,52]. This may emphasize the need for detecting and screening for the elevation in atherogenic risk and predicting IHD in clinical practice. Therefore, this need can be met by utilizing AIP as a clinical tool.

Because high AIP values are associated with a high risk of IHD, persons with high AIP values must strive for lifestyle changes and periodically monitor their lipid profile changes. Lifestyle changes and dietary regimens will also help with weight loss, lowering TG levels, and increasing HDL levels. Pharmacological treatment to control lipid levels can also be considered depending on how an individual’s lipid profile trends. CVD is known to have gender differences in presentation, prevalence, and clinical outcomes [53,54,55]. For instance, men and postmenopausal women showed an increased risk of IHD compared to premenopausal women [56]. It is thought that postmenopausal women have atherogenic LDL profiles comparable to those in men because of aging and the event of menopause [57]. Our study findings also revealed that the higher the AIP quartile, the higher the percentage of men in the highest quartile group. Thus, AIP seems to be a good atherogenic index that is in concordance with the gender differences in IHD. Furthermore, there should be studies evaluating treatment strategies for individuals with high AIP values.

The strength of our study is that we lead a prospective cohort study of Korean adults who were linked to the HIRA data, which were derived from the national insurance coverage system. However, our study also had some limitations. First, the study cohort was comprised of volunteers in a single hospital setting and they visited for the purpose of health promotion screenings. This may have produced a bias, referred to as the healthy volunteer effect. It is worth mentioning that the majority of the patients who visit the hospital are residents of Seoul city, the wealthiest city in South Korea. It is highly likely that the distribution of participants’ economic status and education level is shifted. Thus, there may have been confounding variables that were not considered at baseline. Second, there is a chance that some diabetic patients were accidentally included in the study cohort since glycated hemoglobin A1c and 2-h oral glucose tolerance tests were not initially performed. Thus, this may have influenced the study’s results and interpretation. Third, the shortcoming of the AIP score is that it can have significant variability in different populations [37]. Thus, future investigations are necessary to determine the clinical range of diagnostic AIP values in the Korean population.

## 5. Conclusions

This study identified that elevated AIP is a solid and useful predictor of IHD in the non-diabetic Korean population. In addition, AIP was demonstrated to have good predictive power for IHD compared to isolated TG level or other lipid parameters, in addition to increasing recognition for being a robust and independent value stratifying multiple cardiometabolic risks.

## Figures and Tables

**Table 1:** Baseline characteristics of the study population according to the atherogenic index of plasma (AIP) quartiles.

	AIP Quartiles					
	Q1 *n* = 4529	Q2 *n* = 4358	Q3 *n* = 4582	Q4 *n* = 4475	*p* Value ^1^	Post Hoc ^2^
AIP	≤−0.27	−0.26 to −0.08	−0.07 to 0.14	≥ 0.15		
Age (years)	42.3 ± 10.5	44.6 ± 10.6	46.1 ± 10.4	45.9 ± 10.0	<0.001	a,b,c,d,e
Male sex (%)	24.4	42.4	60.6	76.5	<0.001	-
Body mass index (kg/m^2^)	21.4 ± 2.5	22.6 ± 2.7	23.9 ± 2.8	25.1 ± 2.8	<0.001	a,b,c,d,e,f
Systolic blood pressure (mmHg)	115.4 ± 14.3	119.8 ± 15.1	123.8 ± 14.9	127.8 ± 14.7	<0.001	a,b,c,d,e,f
Diastolic blood pressure (mmHg)	71.7 ± 9.3	74.6 ± 9.8	77.3 ± 9.8	80.1 ± 9.6	<0.001	a,b,c,d,e,f
Mean arterial pressure (mmHg)	86.2 ± 10.6	89.7 ± 11.2	92.8 ± 11.1	96.0 ± 10.9	<0.001	a,b,c,d,e,f
Fasting plasma glucose (mmol/L)	4.9 ± 0.5	5.0 ± 0.5	5.1 ± 0.5	5.2 ± 0.6	<0.001	a,b,c,d,e,f
Total cholesterol (mmol/L)	4.7 ± 0.8	4.8 ± 0.8	5.0 ± 0.9	5.2 ± 0.9	<0.001	a,b,c,d,e,f
Triglyceride (mmol/L)	0.7 ± 0.1	1.0 ± 0.2	1.4 ± 0.3	2.5 ± 1.2	<0.001	a,b,c,d,e,f
HDL-cholesterol (mmol/L)	1.7 ± 0.3	1.5 ± 0.2	1.3 ± 0.2	1.1 ± 0.2	<0.001	a,b,c,d,e,f
C-reactive protein (mg/L)	0.9 ± 2.6	1.3 ± 3.4	1.6 ± 5.1	1.7 ± 3.5	<0.001	a,b,c,d,e
Current smoker (%)	11.0	18.7	28.2	41.3	<0.001	-
Alcohol drinking (%) ^3^	36.1	41.3	45.7	52.5	<0.001	-
Regular exercise (%) ^4^	32.1	32.7	30.1	26.1	<0.001	-
Hypertension (%)	10.0	15.4	22.8	30.5	<0.001	-
Impaired fasting glucose (%)	7.9	13.2	20.0	27.6	<0.001	
Metabolic syndrome (%)	0.6	2.1	7.3	36.1	<0.001	-

^1^*p* values were calculated using one-way ANOVA or Pearson’s chi-square test. ^2^ Post hoc analysis with the Bonferroni method: a, Q1 versus Q2; b, Q1 versus Q3; c, Q1 versus Q4; d, Q2 versus Q3; e, Q2 versus Q4; and f, Q3 versus Q4. ^3^ Alcohol intake ≥ 140 g/week. ^4^ Moderate-intensity physical exercise ≥ three times/week.

**Table 2:** Overall incidence of ischemic heart disease according to the atherogenic index of plasma (AIP) quartiles.

	New Cases of Ischemic Heart Disease (IHD), *n*	Mean Follow-Up, Year	Pearson-Years of Follow-Up	Incidence Rate/1000 Person-Years
Q1	38	2.3 ± 1.1	10,550	3.6
Q2	73	2.4 ± 1.1	10,331	7.1
Q3	98	2.3 ± 1.1	10,752	9.1
Q4	123	2.4 ± 1.1	10,700	11.5

**Table 3:** Univariate Cox proportional-hazards regression model for incident ischemic heart disease (IHD).

	HRs	95% CIs	*p* Value
Age, years	1.071	1.061–1.081	<0.001
Male sex, yes or no	1.614	1.291–2.018	<0.001
Body mass index, kg/m^2^	1.100	1.065–1.137	<0.001
Systolic blood pressure, mmHg	1.017	1.010–1.023	<0.001
Diastolic blood pressure, mmHg	1.025	1.015–1.036	<0.001
Fasting plasma glucose, mg/dL	1.034	1.024–1.045	<0.001
Triglyceride, mg/dL	1.001	1.001–1.002	<0.001
HDL-cholesterol, mg/dL	0.978	0.969–0.987	<0.001
C-reactive protein, mg/L	1.012	0.993–1.031	0.218
Current smoker, yes or no	1.140	0.854–1.521	0.374
Alcohol drinking, yes or no	0.722	0.573–0.909	0.006
Regular exercise^,^ yes or no	1.499	1.193–1.803	<0.001
Atherogenic Index of Plasma (AIP) quartiles, Q1 vs. Q4	3.210	2.231–4.628	<0.001

**Table 4:** Hazard ratios and 95% confidence intervals for new-onset ischemic heart disease (IHD) according to atherogenic index of plasma (AIP) quartiles.

		Q1	Q2	Q3	Q4	*p* for Trend
Model 1	HR (95% CI)	1.00 (reference)	1.63 (1.10–2.42)	1.85 (1.27–2.71)	2.34 (1.61–3.40)	<0.001
	*p* value		0.015	0.001	<0.001	
Model 2	HR (95% CI)	1.00 (reference)	1.59 (1.04–2.45)	1.87 (1.23–2.84)	2.21 (1.45–3.39)	0.003
	*p* value		0.033	0.003	<0.001	
Model 3	HR (95% CI)	1.00 (reference)	1.58 (1.03–2.43)	1.82 (1.20–2.78)	2.11 (1.37–3.24)	0.007
	*p* value		0.037	0.005	<0.001	

Model 1: adjusted for age and sex. Model 2: adjusted for age, sex, body mass index, smoking status, alcohol intake, and physical activity. Model 3: adjusted for age, sex, body mass index, smoking status, alcohol intake, physical activity, mean arterial blood pressure, fasting plasma glucose, high-sensitivity C-reactive protein level, and hypertension medication.

**Table 5:** Hazard ratios and 95% confidence intervals for new-onset ischemic heart disease (IHD) according to atherogenic index of plasma (AIP) quartiles only in the male population.

		Q1	Q2	Q3	Q4	*p* for Trend
Model 1	HR (95% CI)	1.00 (reference)	1.54 (0.85–2.80)	1.88 (1.07–3.30)	2.28 (1.32–3.94)	0.013
	*p* value		0.158	0.027	0.003	
Model 2	HR (95% CI)	1.00 (reference)	1.43 (0.78–2.62)	1.71 (0.96–3.03)	1.89 (1.06–3.37)	0.147
	*p* value		0.244	0.068	0.030	
Model 3	HR (95% CI)	1.00 (reference)	1.43 (0.78–2.62)	1.65 (0.93–2.94)	1.79 (1.00–3.19)	0.238
	*p* value		0.245	0.087	0.049	

Model 1: adjusted for age. Model 2: adjusted for age, body mass index, smoking status, alcohol intake, and physical activity. Model 3: adjusted for age, body mass index, smoking status, alcohol intake, physical activity, mean arterial blood pressure, fasting plasma glucose, high-sensitivity C-reactive protein level, and hypertension medication.

## Data Availability

The data underlying this article will be shared upon reasonable request from the corresponding author.
